# Amphibian-derived wound healing peptides: chemical molecular treasure trove for skin wound treatment

**DOI:** 10.3389/fphar.2023.1120228

**Published:** 2023-06-12

**Authors:** Saige Yin, Ying Wang, Xinwang Yang

**Affiliations:** ^1^ Department of Anatomy and Histology and Embryology, Faculty of Basic Medical Science, Kunming Medical University, Kunming, China; ^2^ Key Laboratory of Chemistry in Ethnic Medicine Resource, State Ethnic Affairs Commission and Ministry of Education, School of Ethno-Medicine and Ethno-Pharmacy, Yunnan Minzu University, Kunming, China

**Keywords:** skin injury, amphibians, wound-healing peptide, bioactive components, ceRNA

## Abstract

Amphibian-derived wound healing peptides thus offer new intervention measures and strategies for skin wound tissue regeneration. As novel drug lead molecules, wound healing peptides can help analyze new mechanisms and discover new drug targets. Previous studies have identified various novel wound healing peptides and analyzed novel mechanisms in wound healing, especially competing endogenous RNAs (ceRNAs) (e.g., inhibition of miR-663a promotes skin repair). In this paper, we review amphibian-derived wound healing peptides, including the acquisition, identification, and activity of peptides, a combination of peptides with other materials, and the analysis of underlying mechanisms, to better understand the characteristics of wound healing peptides and to provide a molecular template for the development of new wound repair drugs.

## 1 Introduction

Skin wound, such as cuts, burns, bites, and other skin wound caused by illness, etc., is a considerable health problem that can cause infection and long-term morbidity and mortality (Zomer and Trentin, 2018; Monavarian et al., 2019). Of note, high-risk patients may develop skin ulcers and may even require amputation due to delayed healing (Aitcheson et al., 2021). Therefore, understanding the mechanisms of skin wound repair is urgent for the development of new repair-promoting drugs (Liu et al., 2019).

The skin of amphibians is directly exposed to different environments and interacts with ambient elements, predators, and microorganisms (Yang et al., 2016; Tong et al., 2019). Given the complex functions of amphibian skin, the need to maintain skin integrity in both aquatic and terrestrial environments, and the fragility of the epidermis, amphibians must protect against skin damage caused by external factors more effectively than other vertebrates (Liu et al., 2021). The bioactive components of amphibian skin secretions, especially bioactive peptides encoded by genes, have been extensively studied in recent decades, showing diverse biological activities, including antibacterial, antioxidant, and repair and renewal activity (Demori et al., 2019; Patocka et al., 2019; Yin et al., 2019). The wound-healing effects of these peptides have received significant attention, possibly due to the fact that scarless repair in amphibians has always been a desirable option for humans (Yokoyama et al., 2018). Therefore, studying wound-healing peptides derived from amphibians deeply can provide valuable information for the development of future wound-healing drugs.

## 2 Mammalian skin structure

The skin is the largest organ in the human body and plays a critical role in protection against the external environment (Khan et al., 2022). Mammalian skin is mainly composed of the epidermis and dermis (Kawasumi et al., 2013; Wong et al., 2015). The epidermis is the superficial layer of the skin and consists of the stratum corneum, granular layer, spinous layer, and basal cell layer (from top to bottom) (Moreci and Lechler, 2020). The keratinocyte layer, primarily composed of keratinocytes, is the outermost layer of the skin and participates in protecting the skin surface (Jiang et al., 2020). The granular layer consists of cells containing flat nuclei and granules (Ishida-Yamamoto et al., 2018), while the spinous layer (also known as the suprabasal cell layer) consists of 5–10 layers of cells connected through prickly structures (Wertz, 2018) and the bottom layer is composed of a single layer of basal cells (Joly-Tonetti et al., 2018). Beneath the epidermis lies the dermis, a highly elastic and flexible tissue composed of collagen, reticular, and elastic fibers and divided into the papillary and reticular layers (Kawasumi et al., 2013). The papillary layer is composed of a variety of cells, including fibroblasts, macrophages, and mast cells, as well as extracellular matrix containing collagen fiber, elastic fiber, and glycoproteins (Yousef et al., 2022). In addition to the elastic fiber network composed of fibroblasts and extracellular matrix, the lower reticular layer also shuttles nerves and blood vessels (Yousef et al., 2022).

## 3 Skin wound healing processes

Skin injury is a complex event that ultimately leads to wound healing (Liu et al., 2019), a dynamic process involving a series of highly overlapping and inter-related stages (Raghavan et al., 2010; Seifert and Maden, 2014; Rodrigues et al., 2019): 1) Hemostasis occurs immediately after wound formation and involves blood component extravasation, platelet aggregation, and blood coagulation to form blood clots that serve as scaffolds for cell migration (Demori et al., 2019). 2) The subsequent inflammatory process encompasses the recruitment of inflammatory cells to the wound site, followed by cytokine release and increased vascular permeability (Cao et al., 2018). During this process, neutrophils, monocytes, and lymphocytes arrive rapidly at the wound site (Li et al., 2018), followed by the secretion of cytokines and chemokines by inflammatory cells to initiate an inflammatory response and facilitate wound repair (Cao et al., 2018). 3) The cell proliferation stage involves the migration and proliferation of keratinocytes, fibroblasts, and endothelial cells, as well as matrix deposition and angiogenesis, leading to re-epithelialization and granulation tissue formation (Gurtner et al., 2008). Keratinocytes and fibroblasts migrate to the wound site, where platelets in the first stage and cytokines in the second stage promote fibroblast and endothelial cell proliferation. These fibroblasts are transformed into myofibroblasts and form granulation tissue at the wound site (Piipponen et al., 2020). 4) In the tissue remodeling stage, the structural integrity and function of the tissue is restored, including the remodeling of the extracellular matrix and scar formation caused by collagen fiber deposition. Interventions at the different stages of wound healing can promote skin wound healing (Wang Y. et al., 2021b).

## 4 Amphibian skin structure

**FIGURE 1:**
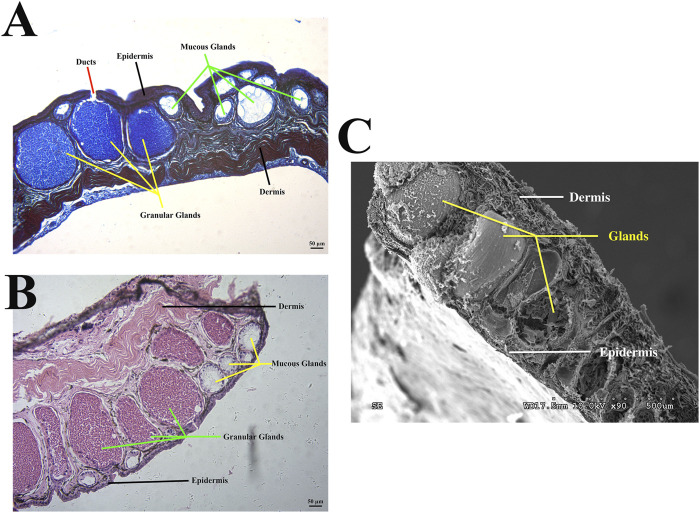
Morphology of *Odorrana andersonii* frog skin. **(A)** Bromophenol blue staining of back skin. **(B)** Hematoxylin and eosin, (H&E) staining of back skin. **(C)** Representative electron microscopy image of back skin.

When amphibians first migrated from water to land as vertebrates, their skin tissue structure also became more adaptable to the environment (Haslam et al., 2014). The structure of amphibian skin tissue is more similar to that of mammals than that of fish. The outer epidermal layer is divided into a cuticle, middle layer, and basal layer (outside to inside), beneath which lies the inner dermal layer composed of a loose layer and dense layer (Yokoyama et al., 2018; Feng et al., 2021) (Figures 1A, B). Skin plays an important role in amphibian survival, including but not limited to respiration, camouflage, water regulation, thermoregulation, excretion, and antimicrobial and antibacterial defense (Schempp et al., 2009). Unlike mammals, amphibians are not protected by hair, so the skin is the first line of defense and plays a prominent role in preventing the loss of vital body fluids, mainly due to dermal glands (Kawasumi et al., 2013; Demori et al., 2019; Liu et al., 2021). Amphibian skin glands are primarily divided into granular and mucous glands. Granular glands exist as a dispersion in the dermis and are the sites of compound formation and release (Barros et al., 2021; Liu et al., 2021) (Figure 1). These glands help resist predator attack by causing pain and aversion, and play an important role in fighting bacterial and microbial infections (Barros et al., 2021). The mucous glands secrete mucus to keep skin smooth and moist, deal with mechanical damage, and inhibit and resist microorganisms (Shi et al., 2020). These glands, dispersed throughout the body, synthesize a variety of bioactive compounds that are secreted via ducts and are essential for survival (Mauricio et al., 2021).

## 5 Characteristic of wound repair in amphibians

**FIGURE 2:**
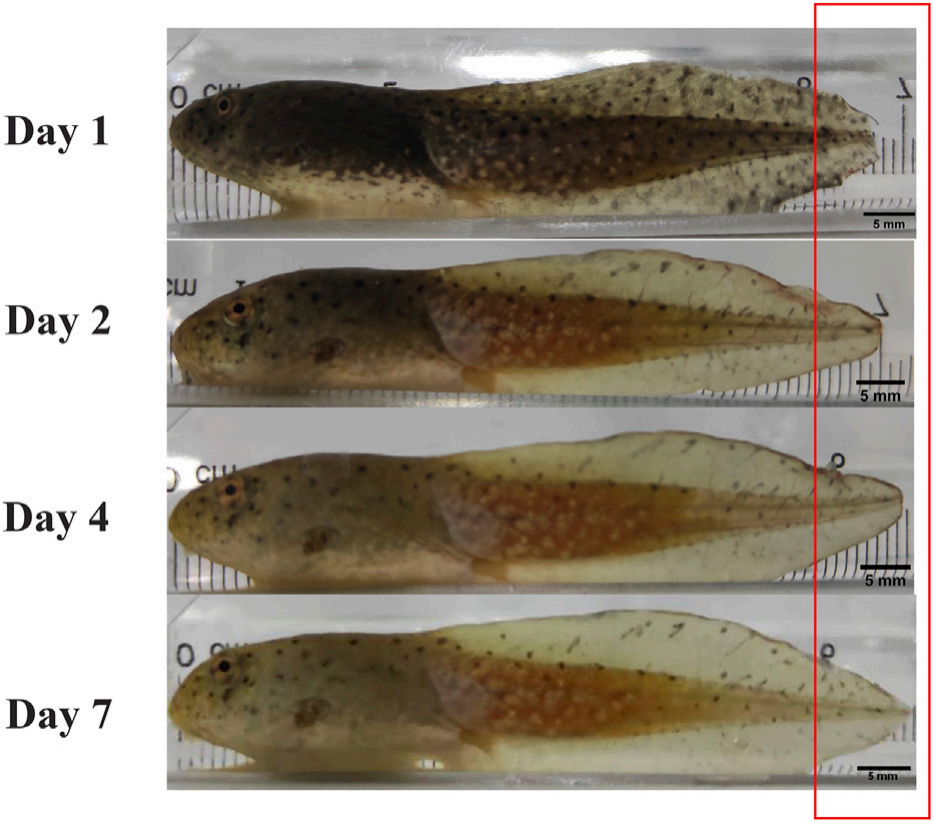
Regenerative ability of amphibians. Regeneration of *Rana catesbeiana* tadpole after tail amputation on days 1, 2, 4, and 7. Red box highlights tail regeneration length.

Although their skin wound repair process is similar to that of mammals, amphibians show a significantly shorter repair time, indicating stronger repair capacity (Levesque et al., 2010; Feng et al., 2021). This may be because amphibian skin healing is not accompanied by scar formation (unlike that in mammals) but rather by regenerative repair (Tseng and Levin, 2008; Demori et al., 2019). As such, amphibian wound re-epithelialization and closure occurs more rapidly (e.g., 2–3 days in mice compared to <10 h in salamanders) and there is better recovery of tissue function and appearance after repair (Figure 2) (Tseng and Levin, 2008; Mu et al., 2014). This rapid skin repair ability is thought to be the long-term process of natural selection. Due to the complexity of their living environments, amphibian skin is extremely vulnerable to external biological and non-biological damage (Feng et al., 2021). As a result, amphibians have evolved a unique skin defense system to ensure survival (Li et al., 2018; Yin et al., 2019; Wang et al., 2022). After proliferation and differentiation, endogenous stem cells develop into mature cells with specific functions that promote tissue regeneration, and amphibian genes also have beneficial effects on wound repair (e.g., *Prx1* gene re-activation in *Xenopus* adults promotes mesenchymal cell proliferation to promote scar-free wound repair, while Tbx5, Fgf8, and Msx1 are also known to participate in amphibian skin wound healing) (Kumar et al., 2004; Yokoyama et al., 2011; Kawasumi et al., 2013; Liu et al., 2014b). Furthermore, bioactive components in amphibians, especially bioactive peptides, play a very important role in skin protection as well (Liu et al., 2014b; Liu et al., 2021). After external skin damage, peptide molecules are released in high concentrations from storage in the granular glands for repair and protection. Thus, amphibian skin is considered to harbor a pool of bioactive molecules with great drug potential (Barros et al., 2021; Wang Y. et al., 2021b). At present, bioactive peptides secreted from amphibian skin include antimicrobial peptides, antioxidant peptides, wound healing peptides, bradykinins, anti-infective peptides, hypoglycemic peptides, neurotoxins, and neuroprotective peptides (Xu and Lai, 2015; Shang et al., 2017; Mwangi et al., 2019; Yang et al., 2019; Yin et al., 2019; Yin et al., 2020). Since tylotoin was first identified in salamander skin in 2014, a growing number of amphibian-derived peptides have been shown to promote wound repair (Mu et al., 2014). However, given the vast treasure trove of bioactive peptides in amphibians, many more remain to be discovered and studied (Mu et al., 2014), which will provide a deeper understanding of these molecules and their potential development.

## 6 Acquisition and identification of wound healing peptides

At present, there are several ways to obtain and identify wound healing peptides.

### 6.1 Separation and purification of peptides

Reverse-phase high-performance liquid chromatography (RP-HPLC) is a useful tool for the separation and analysis of purified peptides. In brief, lyophilized samples are applied to a gel filtration column pre-equilibrated with 25 mm Tris HCl buffer (pH 7.8) containing 0.1 M NaCl, then eluted with the same buffer at a flow rate of 0.1 ml/min. The fractions are then collected by an automatic fraction collector and measured at 280 nm by using a microplate reader. The determined samples are desalted on a C_4_ column by RP-HPLC, then eluted using a C_18_ column. Finally, active peaks are collected (Li et al., 2018).

### 6.2 Primary structure analysis of peptides

Mass spectrometry can be used to analyze the primary structure of peptide molecules, conduct amino acid sequencing of purified peptide molecules, and perform Edman degradation and amino acid residue recognition, thus helping to obtain a complete amino acid sequence (Mu et al., 2014).

### 6.3 Construction and screening of a cDNA library

The construction of a cDNA library involves *in vitro* recombination of cDNA and clone vector DNA, transformation of host cells of clone vector DNA, and procurement of bacterial or phage clones containing recombinant DNA. These sequences represent the entire mRNA population of a certain tissue or cell type at a specific stage of development or differentiation. Given its simple operation and low probability of false positives, this method is widely used to obtain complete nucleotide and encoded amino acid sequences and to clarify their structures to facilitate subsequent synthesis of peptides (Mu et al., 2014; Cao et al., 2018; Song et al., 2019).

### 6.4 Activity tracking of wound-repair peptide molecules

Various experiments can be performed to screen peptide molecules for their wound repair activity, such as calculating cell scratch repair rates, cell proliferation rates, and secretory factor levels after peptide application. The wound healing activity of different peptides can also be assessed by direct application on skin wounds (e.g., whole cortex, scald, and diabetic wound models), with an evaluation of wound healing speed and tissue staining (e.g., H&E and Masson staining) (Liu et al., 2019; Song et al., 2019; Zhang et al., 2022).

## 7 Amphibian-derived wound healing peptides

A growing number of amphibian-derived wound healing peptides have been reported (Li et al., 2018; Demori et al., 2019; Wang Y. et al., 2021b). These peptides, which exist in skin secretions, greatly contribute to skin repair in amphibians (Yokoyama et al., 2018; He et al., 2019). Given the similarity in wound healing between amphibians and humans, we speculate that these active peptides will exhibit similar effects on wound healing in humans (Demori et al., 2019). Thus, these peptide molecules may provide a new template for the development of effective wound-healing drugs. Various amphibian wound healing peptides are described below (Table 1).

**TABLE 1:** Amphibians-derived wound-healing peptides.

Species	Peptide	Sequence (aa)	Length	Molecular mass	Ref.
Wound-healing peptides					
*O. grahami*	AH90	ATAWDFGPHGLLPIRPIRIRPLCG	24	2.6 kDa	Liu et al. (2014b)
*O. tormota*	Ot-WHP	ATAWDLGPHGIRPLRPIRIRPLCG	24	2.6 kDa	He et al. (2019)
*O. andersonii*	OA-GL21	GLLSGHYHRVVSTASGHYGRG	21	2.2 kDa	Bian et al. (2018)
*O. andersonii*	OA-FF10	FFTTSCRSGC	10	1.1 kDa	Liu et al. (2019)
*O. andersonii*	OM-LV20	LVGKLLKGAVGDVCGLLPIC	20	1.9 kDa	Li et al. (2018)
*O. andersonii*	OA-GL12	GLLSGINAEWPC	12	1.3 kDa	Song et al. (2019)
*O. andersonii*	CW49	APFRMGICTTN	11	1.2 kDa	Liu et al. (2014a)
*O. andersonii*	OA-GP11d	GPLSGINAECM	11	2.2 kDa	Fu et al. (2022)
*O. andersonii*	OA-GL17d	GLFKWHPRCGEEQSMWT	17	4.2 kDa	Zhang et al. (2022)
*R. limnocharis*	RL-RL10	RLFKCWKKDS	10	1.3 kDa	Wang et al. (2020a)
*R. limnocharis*	RL-QN15	QNSYADLWCQFHYMC	15	1.9 kDa	Wang et al. (2020b)
*F. cancrivora*	Tiger17	WCKPKPKPRCH-NH2	11	1.4 kDa	Tang et al. (2014)
*B. maxima*	Bm-TFF2	GFPIYEIDNRPGCYVDPAERVACAGAGVTKAECKAKGCCFISARRNTIWCFKLKESADAWKCAVPMNTRVACAGAGVTPAECKGKGCCFNSSYYGTVWCFKPQE	104	-	Zhang et al. (2010)
Antimicrobial peptides with wound-healing activity					
*Salamanders*	Tylotoin	KCVRQNNKRVCK	12	1.5 kDa	Mu et al. (2014)
*Salamanders*	TK-CATH	GGQDTGKEGETGKKKKSDNWFMNLLNKFLELIGLKEAGDDSEPFCFTCIFDMFSQ	55	6.2 kDa	Luo et al. (2021)
*N. ventripunctata*	Cathelicidin-NV	ARGKKECKDDRCRLLMKRGSFSYV	24	2.8 kDa	Wu et al. (2018)
*O. andersonii*	Cathelicidin-OA1	IGRDPTWSHLAASCLKCIFDDLPKTHN	27	3.0 kDa	Cao et al. (2018)
*P. esculentus*	B-2Ta	GILDTLKNLAKTAGKGILKSLVNTASCKLSGQC	33	3.3 kDa	Liu et al. (2017)
*P. lessonae/ridibundus*	Esculenti-1a (1-21) NH2	Esc (1-21), GIFSKLAGKKIKNLLISGLKG-NH2	21	-	Di Grazia et al. (2015)
*D. melanostictus*	Cathelicidin-DM	SSRRKPCKGWLCKLKLRGGYTLIGSATNLNRPTYVRA	37	4.2 kDa	Shi et al. (2020)
*R. temporaria*	Temporins A	FLPLIGRVLSGIL-NH2	13	-	Di Grazia et al. (2014)
*R. temporaria*	Temporins B	LLPIVGNLLKSLL-NH2	13	-	Di Grazia et al. (2014)

### 7.1 Wound-healing peptides

The AH90 peptide (molecular weight 2.6 kDa) identified from the skin of *Odorrana grahami* is composed of 24 amino acid residues and shows significant efficacy in accelerating whole skin wound re-epithelialization and granular tissue contraction in mice (Liu et al., 2014b). The Ot-WHP peptide identified from the Chinese frog *Odorrana tormota* consists of 24 amino acid residues and exhibits 83% similarity with the amino acid sequence of AH90 (He et al., 2019). Ot-WHP also shows significant wound healing effects and a mechanism similar to that of AH90. Both AH90 and ot-WHP can activates the mitogen-activated protein kinase (MAPK) and factor-κB (NF-κB) signaling pathways to induce macrophages to produce chemokines, cytokines, and growth factors and activates the transforming growth factor-β (TGF-β)/Smad signaling pathway to promote cell adhesion and integrin expression to induce keratinocyte migration and fibroblast to myofibroblast transformation, thereby enhancing wound healing. In addition, integrin α5 and α6 expression levels are elevated in keratinocytes after AH90 treatment. Given the cross-talk between integrin and the TGF-β signaling pathway, which also affects TGF-β expression, cell adhesion and migration can be mediated by integrin during skin re-epithelialization, and thus AH90 may promote wound healing speed by up-regulating integrin (Liu et al., 2014b). Ot-WHP can also increase the number of neutrophils and macrophages at the wound site, promote neutrophil phagocytosis, and enhance macrophage, keratinocyte, and fibroblast cross-talk, thus inducing skin wound repair (He et al., 2019). The peptide Bm-TFF2 derived from *Bombina maxima* skin is not only a platelet agonist, but also demonstrated almost an 80% wound closure rate within 36 h *in vitro* scratch experiments on IEC-6 cells (Zhang et al., 2010).

Salamanders are the only adult vertebrates capable of regenerating structurally and functionally intact limbs, signifying excellent wound repair capabilities (Tseng and Levin, 2008; Barros et al., 2021). Tylotoin, the first salamander-derived peptide, induces the formation of endothelial cell tubes, promotes α-SMA expression and angiogenesis, accelerates the transformation of fibroblasts into myofibroblasts, and promotes skin wound healing. In cells, tylotoin induces macrophage migration, stimulates TGF-β1 and Interleukin 6 (IL-6) secretion in macrophages, and activates the Smad and MAPK signaling pathways to regenerate skin tissue (Mu et al., 2014). TK-CATH, another salamander-derived bioactive peptide consisting of 55 amino acid residues, shows similar wound healing activity. Notably, TK-CATH induces macrophages to produce cytokines, growth factors, and chemokines by activating the MAPK signaling pathway and promotes the migration and proliferation of keratinocytes, thereby improving the inflammatory process and tissue remodeling during skin wound healing (Luo et al., 2021).

As a unique amphibian in China (Yin et al., 2020), *O. andersonii* is highly susceptible to skin damage due to the harsh environment in which it lives, thus requiring rapid repair for survival (Bian et al., 2018). As such, *O. andersonii* skin is an important source of potential wound healing peptides, most of these peptides can promote cell scratch repair in a time and dose dependent manner, and exhibit strong healing ability in mouse skin wound models. OA-GL21 (2.2 kDa, 21 amino acid residues) is a bioactive amphibian peptide with wound healing activity but without a disulfide bond or free cysteine residue (Bian et al., 2018). Studies have shown that OA-GL21 Research shows that OA-GL21 can affect cell migration rather than direct proliferation to help wound repair, and form fewer scars during the repair process. Compared with the positive control drug KangFuXin (KFX), OA-GL21 also shows significant effects on mouse skin wounds, with faster and higher healing rates (Bian et al., 2018). The OM-LV20 peptide (1.9 kDa) contains a pair of intramolecular disulfide bonds. In the skin wound healing process, OM-LV20 treatment has a better proliferate effect on HaCaT cells than fibroblasts (Li et al., 2018). OA-GL12, a peptide with one cysteine, may recruit more macrophages to migrate to the wound site by promoting the expression of TNF and TGF-β1 in RAW264.7 cells. In addition, the pronounced free radical scavenging activity of OA-GL12 may also play a role in wound healing (Song et al., 2019). OA-FF10, which is only 10 amino acid residues in length and contains an intramolecular disulfide bridge (*Rana* box), was found to have higher sensitivity to HaCaT cells. This characteristic enables OA-FF10 to accelerate the migration and proliferation of HaCaT cells without affecting HSF cells. Moreover, OA-FF10 (1 μM) also displays better activity than KFX (100 mg/L), thus showing the potential as a wound healing molecule (Liu et al., 2019). The CW49 peptide is very effective in promoting chronic wound healing (Liu et al., 2014a). Chronic diabetic wounds often remain in the inflammatory stage for a long time and inhibiting excessive inflammatory responses can hinder disease progress (Zhao et al., 2016). In diabetic wounds, CW49 application results in significant anti-inflammatory effects (inhibiting IL-6 and TNF-α expression) and angiogenesis (stabilizing HIF-1α expression and up-regulating NO production), thereby preventing excessive inflammatory responses (Liu et al., 2014a). As the first identified natural peptide homodimer shown to promote wound repair, OA-GP11d not only promotes the migration of HaCaT cells but also inhibits the release of inflammatory factors by activating the MAPK and NF-κB signaling pathways. In mouse skin burn and injury models, OA-GP11d also exhibits strong repair ability (Fu et al., 2022). The other natural repair-promoting homodimer peptide OA-GL17d (OA-GL17 dimer) has a half-life of 1.86 h (longer than some peptides), this means that it may have more advantages in the effective time than other similar wound-healing peptides. OA-GL17d shows strong repair ability in many animal wound models (e.g., mouse whole skin and scald wound models). This repair effect may occur by reducing miR-663 levels, increasing TGF-β1 levels, activating the TGF-β1/Smad signaling pathway, and ultimately accelerating re-epithelialization and granular tissue formation in skin wounds (Zhang et al., 2022).

Despite its wide distribution in Southeast Asia, little research has been conducted on the *Rana limnocharis* frog species and its peptides (Sumida et al., 2002). RL-RL10, an active peptide molecule with a molecular weight of only 1.3 kDa, has been shown to improve the proliferation and migration of HaCaT cells in a concentration-dependent manner, and enhance full-thickness wound healing in mice (Wang S. et al., 2021a). RL-QN15, another short peptide derived from skin secretions of *R. limnocharis*, also shows potent repair effects in various wound models (chronic wounds, skin fibrosis, and oral ulcers). RL-QN15 can induce keratinocyte migration and proliferation, which are crucial for initial wound healing. RL-QN15 can also significantly inhibit the pro-inflammatory factor TNF-α and promote IL-1β to recruit macrophage migration to the wound. During wound healing, excessive inflammation can lead to scar formation, thus inhibiting TNF-α levels may contribute to scar reduction (Kumar and Yin, 2018). In addition, different release levels of TGF-β1 and TGF-α in different periods caused by RL-QN15, can help balance the TGF-β1 and TGF-α ratio during wound repair, which is also one of the reasons for faster wound healing and reduction of scar formation. This above phenomenon may be caused by the activation of the MAPK and Smad signaling pathways in wound skin (Wang Y. et al., 2021b). RL-QN15 also shows improved wound healing ability in combination with nanomaterials, as discussed later in this review.

### 7.2 Antimicrobial peptides with wound-healing activity

In the process of wound healing, endogenous infection caused by microorganisms can directly damage wound epithelialization, which is an important factor hindering wound repair (Ki and Rotstein, 2008). Therefore, enhancing the antibacterial effects of skin during wound infection should promote the healing speed of skin wounds.

The cathelicidin-NV peptide from the plateau frog *Nanorana ventripunctata* belongs to the cathelicidin family and consists of 24 amino acid residues (Wu et al., 2018). The cathelicidin family, which only participates in vertebrate humoral immunity, is an antibacterial and immunostimulatory family and plays an important role in immunoregulation, wound healing, and angiogenesis (Cao et al., 2018). Peptide tylotoin, TK-CATH (both derived from salamanders), and cathelicidin-OA1 (isolated from *O. andersonii*) also belong to this family (Mu et al., 2014; Liu et al., 2019; Luo et al., 2021). As the first frog species-derived peptide to show wound healing activity (Cao et al., 2018), cathelicidin-OA1 also exhibits antioxidant activity. Because the oxidative stress is detrimental to wound repair, so the healing effects of cathelicidin-OA1 are achieved not only by direct intervention in the wound healing process but also via antioxidant activity (Cao et al., 2018). Local application of cathelicidin-NV in whole skin wounds promotes wound re-epithelialization and accelerates healing. Cathelicidin-NV can also induce fibroblasts to produce collagen and promote keratinocyte proliferation to facilitate granulation tissue formation. Furthermore, cathelicidin-NV treatment can increase the levels of Monocyte chemoattractant protein-1 (MCP-1), tumor necrosis factor, -α (TNF-α), vascular endothelial growth factor (VEGF), and TGF-β1 in skin, which is favorable for the formation of blood vessels (Park et al., 2017; Wu et al., 2018).

Peptide B-2Ta, identified from the European frog *Pelophylax kl. esculentus*, is an effective antibacterial peptide. B-2Ta administration suppresses the inflammatory response and promotes angiogenesis in injured rats. Treatment of infected granulation tissue with B-2Ta inhibits inflammation of injured tissue, promotes angiogenesis and epithelial migration, and accelerates wound healing. Thus, B-2Ta is both an antimicrobial and wound healing peptide (Liu et al., 2017). The antimicrobial peptide fragment Esculenti-1a (1-21) NH2, isolated from the skin of *Pelophylax lessonae/ridibundus*, also promotes wound healing, especially in chronic skin ulcers. *In vitro*, Esculenti-1a (1-21) NH2 promotes HaCaT cell and primary epidermal keratinocyte migration to enhance wound epithelialization. In mice, Esculenti-1a (1-21) NH2 releases TGF-β1 and activates the EGFR signaling pathway to promote wound repair (Di Grazia et al., 2015). Temporins A and B, antimicrobial peptides derived from *Rana temporaria*, also exhibit skin wound healing activity. Notably, temporins A and B can reduce *Staphylococcus aureus* in HaCaT cells in a dose-dependent manner to induce cell proliferation, thereby promoting keratinocytes to initiate wound closure. These peptides can also induce HaCaT cell migration to the wound by activating the EGFR signaling pathway (Di Grazia et al., 2014). Cathelicidin-DM, an antibacterial peptide derived from *Duttaphrynus melanostictus*, also has wound healing properties. In mice, cathelicidin-DM markedly accelerates skin infection wound healing, with a stronger effect than that of the aminoglycoside antibiotic gentamicin (Shi et al., 2020).

## 8 Future of wound healing peptides: experiments to clinics

Given the global aging population, the occurrence of accidents and sequelae of various diseases such as diabetes will likely lead to an increase in acute and chronic wounds (Cao et al., 2018; Li et al., 2018; Bai et al., 2020). Wound repair is a highly complex process, thus interfering with different stages of wound healing, such as reducing inflammation, enhancing cell proliferation, and improving tissue remodeling, is an effective way to promote wound repair (Pazyar et al., 2014). At present, clinical wound treatment primarily relies on growth factor drugs such as VEGF and erythropoietin, which have high production, storage, and transportation costs and may exhibit tumor promoting effects with continuous use, and thus fail to achieve ideal therapeutic effects (Hamed et al., 2010; Julier et al., 2017; Cao et al., 2018). Furthermore, existing drugs are insufficient compared to the huge clinical demand. Therefore, how to heal wounds effectively and quickly and how to develop new wound healing drugs remain hot issues in the scientific community.

Compared with conventional chemical drugs, peptides show higher specificity, safety, and efficiency, but are not easily accumulated in the body, making them attractive in the development of new drugs (Hancock et al., 2016; Zhao et al., 2022). However, peptides also have some disadvantages, such as unstable chemical properties, short half-life, fast clearance, and easy enzymatic hydrolysis (Fosgerau and Hoffmann, 2015; Lee A. C. et al., 2019a). Despite their high bioactivity, short sequences, and ease of synthesis, there is still a long way to go before the clinical use of peptides (Lee A. C. et al., 2019a), although many studies have explored how to better use and promote their application.

### 8.1 Structural modification of existing peptides

Modification of peptides can not only improve peptide activity but also reduce production costs. Peptide molecules are generally composed of several to dozens of amino acid residues (Bolhassani, 2019; Sun Z. G. et al., 2021b) and can be transformed by directly modifying the peptide chain skeleton, shortening the length of the peptide chain skeleton, and intercepting the final effective peptide segment.

### 8.2 Synthesis and screening of new peptide molecules based on existing peptides

Tiger17, an 11 amino acid residue peptide synthesized base on peptides tigerinins (from the skin secretions of *Fejervarya cancrivora*), shows potent skin wound healing activity in mice. This promotion ability may occur by inducing macrophages to re-aggregate to the wound site and promoting keratinocyte and fibroblast migration and proliferation, leading to re-epithelialization and granulation tissue formation, activation of the MAPK signaling pathway, and transfer of TGF-β1 and IL-6 in macrophages. Compared with basic peptide molecules, these peptides tend to have shorter sequences, higher activity, and wider application potential (Tang et al., 2014).

### 8.3 Combining peptides and emerging materials

#### 8.3.1 Peptides combined with nanomaterials

Nanomaterials are important therapeutic agent carriers in wound therapy (Ashraf et al., 2016). Loading therapeutic molecules into nanomaterials for continuous release at the application site can significantly improve therapeutic efficacy (Huh and Kwon, 2011). For example, RL-QN15 shows higher wound repair activity when combined with HPDA nanoparticles (HPDAIR) and mesoporous polydopamine (MPDA) nanoparticles than when used alone. Notably, these peptide nanomaterial composites show improved regeneration-promoting ability in mouse full-thickness skin wound and rat oral ulcer wound models, as well as increased wound healing activity in mouse scald and porcine full-thickness skin wound models (Sun H. et al., 2021a; Qin et al., 2021). Furthermore, compared to the application of the peptide alone, Esculen-tin-1a (1-21) NH2 composites [synthesis: AuNPs@Esc (1-21)] not only inhibit *Pseudomonas aeruginosa*, but also increase re-epithelialization activity of keratinocytes to help accelerate the healing of chronically infected wounds (Casciaro et al., 2017).

#### 8.3.2 Peptides combined with other materials for wound dressings

Wound dressings are important interventions to promote and accelerate wound healing, reduce scar formation, and inhibit microbial invasion (Salehi et al., 2013; Morgado et al., 2015). The binding of the RL-QN15 peptide with hollow silica nanoparticles (HSN) and zinc alginate (ZA) gel to form an HSN@RL-QN15/Za hydrogel promotes cell proliferation and keratinocyte scratch repair, regulates angiogenesis, reduces inflammation, accelerates skin re-epithelialization and granulation tissue formation, ultimately leading to the rapid healing of full-thickness skin wounds and methicillin-resistant *Staphylococcus aureus* biofilm-infected wounds in mice (Qin et al., 2022). Furthermore, combining the RCSP peptide (derived from *R. limnocharis* skin) with electrospun poly (L-lactide)/zein nanofiber mats can significantly improve the mechanical properties of the mats and ameliorate cell survival, adhesion, and proliferation rates, suggesting potential as an ideal wound dressing (Zhang et al., 2016). Similarly, coaxial electrospinning of RCSP and sodium alginate (SA) to form composite nanofiber SA@Ca^2+^/Rcsps gel can promote collagen deposition, rapid wound hemostasis, and epidermal regeneration (Li et al., 2019).

## 9 Peptides as molecular probes for analyzing skin wound repair mechanisms

**FIGURE 3:**
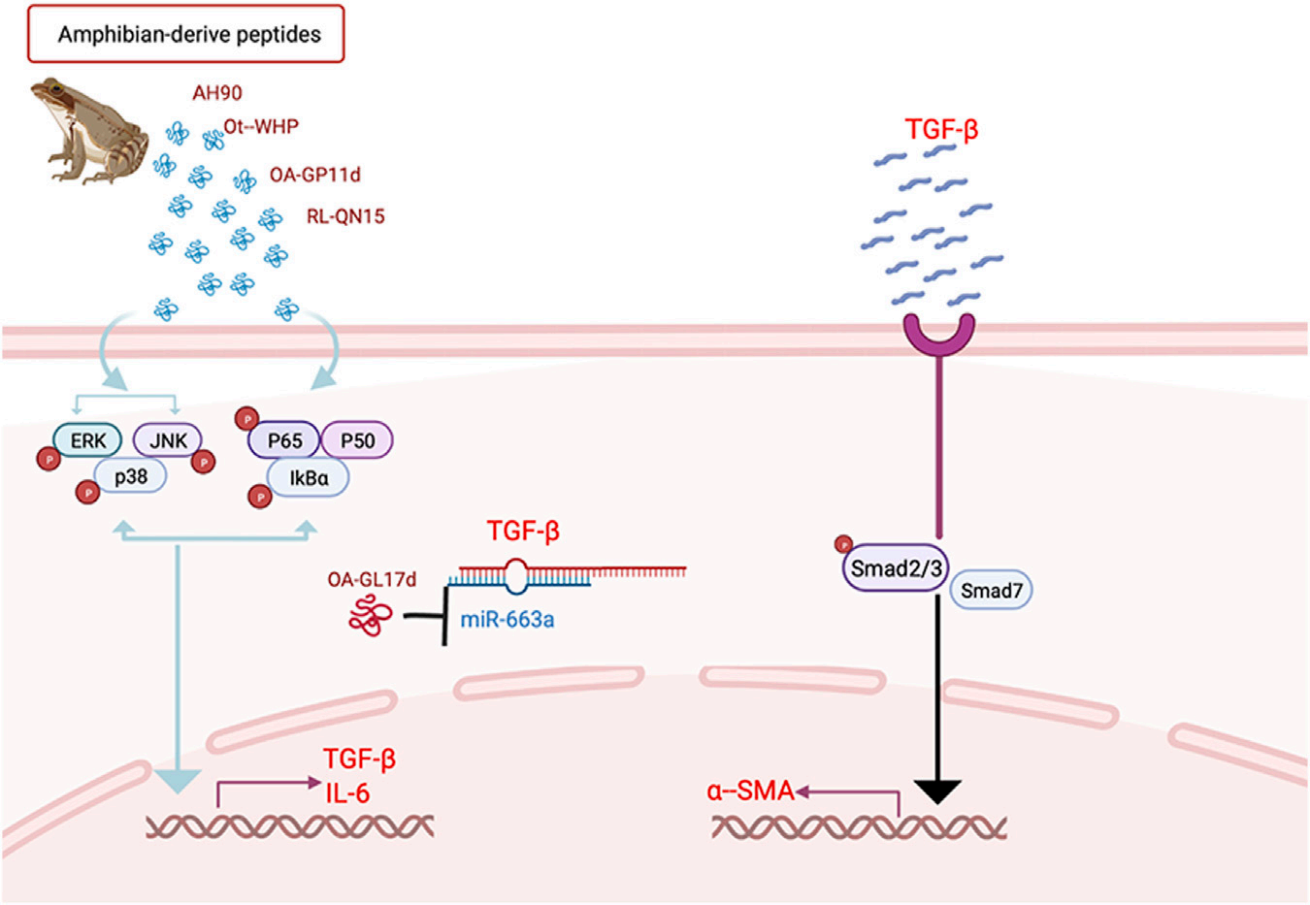
Common signaling mechanisms of Amphibian-derived wound healing peptides.

As molecular probes, amphibian-derived peptides have become an important link in helping to understand the wound healing process and elucidate related mechanisms (Zhang et al., 2022). Based on the above peptides, most wound repair mechanisms appear to be related to the activation of various signaling pathways, such as the TGF-β1/SMAD, MAPK, and NF-κB signaling pathways (Liu et al., 2014b; Wang Y. et al., 2020b; Fu et al., 2022), although the importance of competitive endogenous RNA (ceRNA) in skin wound repair is also emerging (Zhang et al., 2022) (Figure 3).

### 9.1 Activation of signaling pathways

TGF-β is an important growth factor in the body and is essential for wound healing, particular the TGF-β1, 2, and 3 subtypes (Lichtman et al., 2016). TGF-β1 is often released during the acute reaction period of trauma, which helps macrophages and fibroblasts to become chemotactic towards the wound and promotes keratinocyte proliferation (Peplow and Chatterjee, 2013). TGF-β 3 also shows an important influence on cell migration regulation (Le et al., 2012). SMAD is the downstream effector of TGF-β, and TGF-β/SMAD signaling pathway activation can increase skin angiogenesis, promote wound contraction, and inhibit inflammation by inducing fibroblast transformation and integrin expression (Wang Y. et al., 2021b). The MAPK and NF-κB signaling pathways form the intersection of various signaling pathways in wound repair. They are closely related to inflammation and inhibiting their phosphorylation can help reduce inflammatory factor levels and inflammation during wound healing (Fu et al., 2022). Furthermore, studies have identified cross-talk between the TGF-β and MAPK signaling pathways (Liu et al., 2014b).

### 9.2 Mechanism of ceRNAs

The ceRNA mechanism has attracted considerable attention and has helped to reveal the interactions between RNAs. Notably, ceRNAs can change the expression of target genes by competing for shared microRNAs (miRNAs) at the post-transcriptional level (Wu et al., 2020). In the human genome, most DNA is transcribed into RNA to become non-coding RNA, which cannot encode proteins. Among them, miRNAs play a variety of important regulatory roles in cells, including repression of target protein translation (Lu and Rothenberg, 2018). In recent years, the ceRNA mechanism has been implicated in many diseases, implying important biological significance. As miRNAs participate in almost all stages of wound healing, they are considered important targets in the intervention of skin wound healing. For example, miR203 is associated with the promotion of keratinocyte proliferation and migration (Liu et al., 2022), and miR19a and 20a are involved in the activation of the NF-κB signaling pathway (Li et al., 2021). However, although certain miRNAs appear to be involved in wound healing, research remains poor in comparison to the huge miRNA family, and exploration of miRNA functions and the ceRNA mechanism is also limited. As mentioned above, miR-663a is the first wound healing miRNA identified using an amphibian-derived peptide (OA-GL17d) as a molecular probe. Inhibition of miR-663a can improve TGF-β levels and activate the TGF-β/SMAD signaling pathway, thereby accelerating wound repair (Zhang et al., 2022). Thus, these findings suggest that ceRNAs are important for a deeper understanding of the mechanisms underpinning wound healing and emphasize the importance of peptides as molecular probes to analyze such mechanisms.

## 10 Discussion and conclusion

**FIGURE 4:**
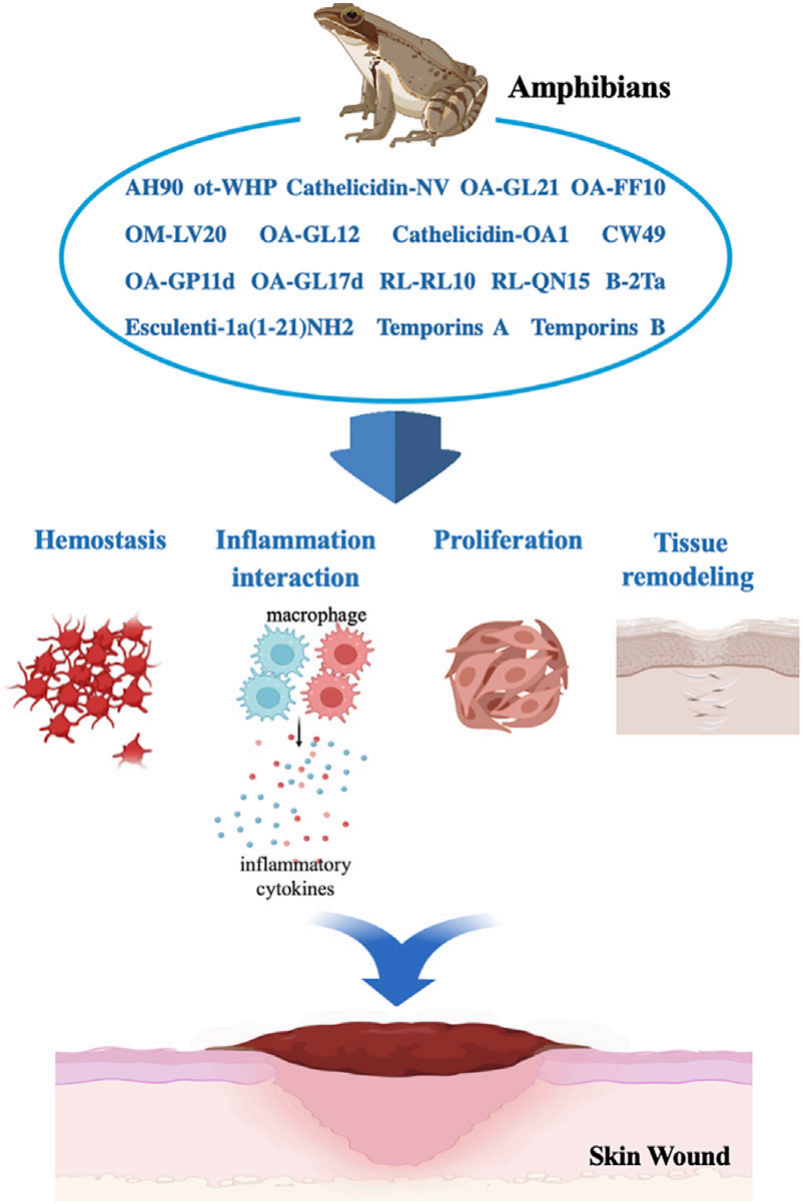
Benefit effects of amphibian-derived wound-healing peptides in skin wound repair process.

Amphibians are an important source of peptide molecules (Yang et al., 2016). These peptides are not only expected to become new drug candidates for treatment but also molecular probes to analyze various mechanisms underlying human diseases. As an exogenous molecular probe, OA-GL17d has been used to explore and analyze the importance of the ceRNA mechanism, while nerve growth factor (NGF) derived from snake venom has been used to clarify related diseases, such as pheochromocytoma and hereditary sensory neuropathy (Yang et al., 2019; Zhang et al., 2022). Although a number of natural wound healing peptides have been explored, our understanding, discovery, and development of amphibian-derived peptides remain limited, especially given the huge drug resource pool with development potential (Figure 4), (Cao et al., 2018; Song et al., 2019). At present, 20 peptide-based clinical trials currently underway conducted, with more than 400 peptide drugs developed each year. Although the potential application value of peptides, especially those from amphibians, is increasingly recognized (Lee A. C. et al., 2019a; Lee C. L. et al., 2019b), based on our knowledge, there are still don't have current or completed clinical trials on amphibian-derived wound-healing peptides. The exploration of amphibian wound repair-promoting peptides and related mechanisms requires further research, and the peptides are also believed to have potential for clinical application.
